# Antiplasmodial, Antitrypanosomal, and Cytotoxic Effects of *Anthonotha macrophylla*, *Annickia polycarpa*, *Tieghemella heckelii,* and *Antrocaryon micraster* Extracts

**DOI:** 10.1155/2022/9195753

**Published:** 2022-07-23

**Authors:** Aboagye Kwarteng Dofuor, Emmanuel Kofi Kumatia, Jersley Didewurah Chirawurah, Frederick Ayertey

**Affiliations:** ^1^Department of Biological, Physical and Mathematical Sciences, University of Environment and Sustainable Development, Somanya, Ghana; ^2^Department of Phytochemistry, Centre for Plant Medicine Research, Mampong-Akwapim, Ghana; ^3^Department of Biochemistry Cell and Molecular Biology, University of Ghana, Legon, Ghana

## Abstract

Malaria and trypanosomiasis are protozoan diseases which pose a devastating challenge to human health and productivity especially, in Africa where their respective vectors (female *Anopheles* mosquito and tsetse fly) abound. Various medicinal plants are used to treat these parasitic diseases. However, the scientific basis of their use and toxicological profiles have not been assessed. We have, therefore, evaluated the antiplasmodial, antitrypanosomal, and cytotoxic activities of four African medicinal plant extracts namely, *Anthonotha macrophylla* leaf (AML), *Annickia polycarpa* leaf (APLE), *Tieghemella heckelii* stem bark (THBE), and *Antrocaryon micraster* stem bark (AMSBE) extracts in vitro against *P. falciparum* (W2mef laboratory strain), *T. brucei* (GUTat 3.1 strain), and mammalian RAW 264.7 macrophage cell line, respectively. The most active antiplasmodial extract was AML (IC_50_ = 5.0 ± 0.08 *μ*g/mL with SI of 21.9). THBE also, produced the most effective antitrypanosomal activity (IC_50_ = 11.0 ± 0.09 *μ*g/mL and SI of 10.2) among the extracts. In addition, none of the extracts produced toxic effect in the RAW 264.7 macrophage cell line except APLE which was moderately cytotoxic and also produced the least SI in both antitrypanosomal and antiplasmodial assays. These results suggest that AML and THBE could offer safe and alternative therapy for malaria and trypanosomiasis. This is the first study to report the antitrypanosomal and in vitro antiplasmodial activities of these four plants/plant parts. The cytotoxicity of the plant parts used is also being reported for the first time except for the *T. heckelii* stem bark.

## 1. Introduction

Malaria is caused by several species of protozoan parasites from the genus *Plasmodium*. In sub-Saharan Africa, the most pathogenic species is the *Plasmodium falciparum* that causes more than 90% of all malaria cases [1]. The World Malaria Report reported estimated 241 million malaria infections and 627 000 malaria deaths globally in 2020, which denotes about a 14 million increase in cases and 69 000 additional deaths, in 2020 as compared to 2019 [1]. Even though the development of resistance, by the *Plasmodium* parasite, to artemisinin and its combination therapies (ACTs) in sub-Saharan Africa is slower than that in places like Asia and South America, the burden of malaria in the former region remains high [1–3].

African trypanosomiasis, a tsetse-transmitted disease of humans and livestock caused by protozoan parasites of the genus *Trypanosoma*, is also of serious health and economic concern in various sub-Saharan African countries [4, 5]. In the absence of vaccines, chemotherapy remains the only practical means to control African trypanosomes [6]. However, development of resistance to present drugs, side effects, and difficulty in regimen application pose serious challenges to chemotherapy [7–10].

Macrophages are mononuclear phagocytes that form critical components of the mammalian immune system. RAW cells are macrophages originally established from an ascitic tumor induced in a male BALB/c mouse by intraperitoneal injection of the Abelson leukemia virus [11]. RAW 264.7 cell lines are useful in the evaluation of the bioactivity of natural products as well as envisage their probable in vivo outcomes [12] and toxicity. Thus, they provide useful advantages in drug discovery research.

Folkloric medicinal plants have proven to be the most versatile source of crude and purified drugs for mankind [13]. Furthermore, it has been documented that approximately 60% of pharmaceutical drugs being used currently were directly or indirectly derived from plants and other natural origins [14]. Due to the challenges associated with current antimalarial and antitrypanosomal drugs, the need to find alternate and new medications to combat these diseases, especially, from folkloric medicinal plants has become a focus of the global scientific research.

Four antimalaria folkloric African medicinal plants have been selected for this study. Malaria and trypanosomiasis are both protozoan diseases. Hence, the extracts were tested against both parasites. The selected medicinal plants are discussed below.

*Anthonotha macrophylla* (P. Beauv), Caesalpiniaceae, is an evergreen tree [15] whose leaf is a remedy for diarrhoea, dysentery, skin infections, headache, and obnoxious stings [16–18]. The stem bark is used to treat venereal diseases and malaria. So far, aphrodisiac, safety, anti-inflammatory, analgesic, and antioxidant activities of the leaf in addition to antiplasmodial activity of the stem bark have been reported [15, 19–21]. *Antrocaryon micraster* (A. Chev. and Guillaumin), Anacardiaceae, is a timber species, whose stem bark is used to treat malaria, impotence, bodily pains, and arthritis [22–24]. Anti-inflammatory, antioxidant and in vivo antimalaria activities were reported for the plant [25, 26].

*Annickia polycarpa* ((DC.) Setten and Maas ex I.M. Turner.), a member of the Annonaceae family, is employed to treat malaria, fever, stomach ulcer, pyrexia, bacterial infections, injuries, eye infections, and wounds [27–30]. Antimalaria, antitrypanosome, analgesic, anti-inflammatory, and antibacterial activities have been reported [26–31]. The stem bark of *Tieghemella heckelii* ((A. Chev.) Pierre ex. Dubard), a timber species of the Sapotaceae family, is used to treat toothache, blennorrhoea, and malaria, whereas the seed is employed against hernia [32–34.]. The stem bark of *T. heckelii* has been reported to possess cytotoxic, antibacterial, analgesic, and anti-inflammatory activities of the plant [33, 35–37].

Although the in vivo antimalaria activity of *A. micraster* stem bark*, A. polycarpa* stem bark, and leaf in addition to the in vitro antimalaria activity of the *A. macrophylla* stem bark have been reported, the antitrypanosomal activity of the four plants and/or plant parts is not known. In addition, the in vitro antiplasmodial activity and the selectivity index (SI) of the selected plant parts have also not been reported. This study, therefore, sought to evaluate the in vitro antiplasmodial, antitrypanosomal, and cytotoxic activities of the *A. macrophylla* leaf (AML), *A. micraster* stem bark (AMSBE)*, A. polycarpa* leaf (APLE), and *T. heckelii* stem bark (THBE).

## 2. Materials and Methods

### 2.1. Chemicals and Reagents

HMI9, FBS, and DMEM were purchased from Thermo Fisher Scientific, UK. Other chemicals and reagents such as penicillin-streptomycin-L-glutamine (PSG), 2-[4-(2-hydroxyethyl) piperazin-1-yl] ethanesulfonic acid (HEPES), chloroquine, diminazene aceturate, Alamar dye, SYBR Green I, dimethyl sulphoxide (DMSO), sodium citrate, adenine, sodium bicarbonate (NaHCO_3_), sodium chloride (NaCl), sodium phosphate dibasic (Na_2_HPO_4_), potassium chloride (KCl), sodium phosphate monobasic (KH_2_PO_4_), sodium hydroxide (NaOH), and sodium bicarbonate (NaHCO_3_) were also purchased from Sigma-Aldrich, UK.

### 2.2. Identification, Collection, Processing, and Extraction of Plant Materials

The plants were identified and collected by Mr. Jonathan Dabo, a botanist at the Forestry Research Institute of Ghana (FORIG), Kumasi. The *A. macrophylla* leaf, *A. polycarpa* leaf, and *T. heckelii* stem bark were collected from the Bobiri Forest Reserve in the Ashanti Region of Ghana. The *A. micraster* stem bark was collected from the Asubima Forest Reserve in the Ahafo Region of Ghana. *A. polycarpa* leaf, *T. heckelii,* and *A. micraster* stem barks were assigned voucher specimen numbers (FORIG 0012, FORIG 0013, and FORIG 0014, respectively), and deposited at the herbarium of FORIG. *A. macrophylla* leaf was given a voucher specimen number of CPMR 4937 at the herbarium of the Centre for Plant Medicine Research, Mampong-Akwapim, Ghana. The plant materials were sun dried for 8 days and pulverized into a coarse powder. Extraction was performed using the protocol of Kumatia et al. [20]. Briefly, the powdered stem bark of *A. micraster* (200 g) was extracted with 70% ethanol (2 L × 2) at room temperature for four days each filtered and combined. Ethanol in the extract was removed by concentrating the filtrate at low pressure in a rotary evaporator (Eyeler N1110, Tokyo-Japan). The aqueous part was lyophilized to obtain a powder- coded AMSBE. A similar procedure was applied to the other plant materials to obtain solid extracts coded APLE, THBE, and AML for *A. polycarpa* leaf, *T. heckelii* stem barks, and *A. macrophylla* leaf, respectively. The extracts were stored in air-proof containers at 4°C until required.

### 2.3. Phytochemical Screening of the Extracts

An aliquot of each extract (50 mL) was analyzed for the presence or absence of phytochemical compounds such as flavonoids, alkaloids, triterpenes, phenolic compounds, saponins, polyuronoids, antracenosides, reducing sugars, phytosterols, and cyanogenic glycosides as per the methods described by Fong et al. [38].

### 2.4. Culturing of Plasmodium falciparum

W2mef laboratory strain of *P. falciparum* was cultured with human group O^+^ erythrocytes using standard methods [39] with slight modifications. *P. falciparum* parasites were cultured to >5% parasitemia of ring-stage parasites. Using 5% sorbitol treatment, a synchronized culture of ring-stage parasites [40] was obtained and diluted to 1% parasitemia and 2% hematocrit for the growth inhibition assays.

### 2.5. Evaluation of Antiplasmodial Activity

Extracts were tested against P. falciparum activity in the SYBR Green I fluorescence assay [41]. Extracts at concentrations of 10 mg/mL were diluted with culture media to a starting working concentration of 100 *µ*g/mL. Serial dilutions (1 : 2) were made to yield 7 final concentrations of each of the extracts (100–0.78 *µ*g/mL). An aliquot of 10 *µ*L of each concentration was dispensed into test wells of the 96-well plate in triplicates with each tested well already containing 90 *µ*L of 2% hematocrit and 1% parasitemia. Wells containing RBCs at 2% hematocrit and 1% parasitemia were used. The final volume per well was 100 *µ*L. Plates were incubated for 48 h, and an aliquot of 100 *µ*L of 4x buffered SYBR Green I (0.25 *µ*L of 10000x SYBR Green I/mL of 1x phosphate buffer saline) was added to each well after the incubation period and incubated again in the dark for 30 min at 37°C. The presence and the amount of infected red blood cells (RBCs) were detected using the BD FACS LSRFortessa™ X-20 flow cytometer and analyzed with BD FACSDiva Software (v8.0.1). A total of 5,000 RBCs were counted to determine the number of infected RBCs (SYBR Green I positive cells) present. Chloroquine (CHL) was used as the reference antiplasmodial drug.

### 2.6. Culturing of Trypanosoma brucei Parasites

Bloodstream forms of the subspecies *T. brucei* (GUTat 3.1 strains) were cultured *in vitro* to the logarithm phase using Hirumi's modified Iscove's medium (HMI9, Thermo Fisher Scientific) with 10% foetal bovine serum (Thermo Fisher Scientific) at 5% CO_2_ and 37°C using manufucturer's instruction.

### 2.7. Evaluation of Antitrypanosomal Activity

The assay was performed as described previously [42]. Cells were seeded at a density of 3.0 × 10^5^ cells/ml in 96-well plates in a two-fold dilution of extracts. Extracts were incubated in a two-fold dilution with the cells for another 24 h. Alamar blue dye (resazurin, 10% v/v) was then added to all wells and incubated for another 24 h. Spectrophotometric absorbance was recorded at a wavelength of 570 nm. Diminazene aceturate (DA) was used as a positive antitrypanosomal control drug.

### 2.8. Culturing of Mouse Macrophages (RAW 264.7) Cell Line

Mouse macrophages (RAW 264.7 cell lines) were cultivated *in vitro* to the logarithm phase using Dulbecco's modified Eagle media (DMEM, Thermo Fisher Scientific) with 10% foetal bovine serum at 5% CO_2_ and 37°C.

### 2.9. Evaluation of Cytotoxity of the Extracts against Mouse Macrophages (RAW 264.7) Cell Line

Cell lines were plated at a density of 3.0 × 10^5^ cells/mL for 48 h to allow for sufficient adherence to plates. Extracts were incubated in a two-fold dilution with the cells for another 24 h. Alamar blue dye (resazurin, 10% v/v) was then added to all wells and incubated for another 24 h to allow for a complete color development. Spectrophotometric absorbance was recorded at a wavelength of 570 nm.

### 2.10. Selectivity Index (SI)

The SI was calculated for each extract in each test to determine their effectiveness and toxicity in their use as an antiplasmodial or antitrypanosomal agent using the following formula:

(1) $$\begin{array}{c} \text{SI}=\frac{\text{IC}50\text{ obtained for the extract in the RAW macrophage cell}}{\text{IC}50\text{ obtained for the extract against the protozoan parasite}}. \end{array}$$

### 2.11. Statistical Analysis

Algorithms obtained from flow cytometry (FACS) were analyzed using regression equations of best fit of plotted growth inhibition versus concentration curves. GraphPad Prism for Windows version 7.0 (Graph Pad Software, San Diego, CA, USA) was used for all statistical analyzes. Cell viability of parasites was estimated from the fluorescence of SYBR Green I or absorbance of resazurin and represented as a percentage of treated to untreated cells. IC_50_ values were calculated from a nonlinear regression model. Data were presented as the mean ± S.E.M.

## 3. Results and Discussion

### 3.1. Yield and Nature of Crude Extracts

The weight, yield, and nature of the extracts obtained are described in Table 1. The result shows that the stem barks produced higher yields of extracts than the leaves.

The stem bark extracts yielded more extracts than the leaves under the same conditions. This was due to the fact that most trees have very thick and malleable stem barks. THBE produced the highest yield of the extract (21.88% w/w). This was followed closely by AMSBE. AML produced the least yield of the extract (4.72% w/w). This indicates that less plant material is needed to produce more extracts for the plant parts that gave high yield and vice versa.

### 3.2. Classes of Phytochemical Constituents in the Extracts

The results of the phytochemical analysis of the extracts are tabulated in Table 2. The results show that all the extracts contained saponins, phenolics, and reducing sugars. Phytosterols were found in all the extracts except AMSBE. AML and THBE also contained flavonoids in addition to the other four constituents. Alkaloids were also present in APLE, which contained a greater number of phytochemical constituents among the extracts.

The yield, nature, and phytoconstituents obtained for the extracts were similar to those reported elsewhere [20, 26, 31] because it was the same bath of the plant material used in both studies.

### 3.3. Antiplasmodial, Antitrypanosomal, and Cytotoxic Activities

The results of the in vitro antiplasmodial, antitrypanosomal, and cytotoxic activities of the extracts are shown in Table 3.

The extracts produced antiplasmodial activity with IC_50s_ of 5.0 ± 0.08–110.1 ± 0.12 *μ*g/mL and that of CHL was 0.146 ± 0.05 *μ*g/mL. AML was the most active extract. The extracts can be arranged in increasing order of antiplasmodial activity as follows: APLE < AMSBE < THBE < AML. According to Zirihi et al. [15], the in vitro antiplasmodial activity of the plant extracts is classified as good, weak, or inactive using their IC_50_ values. The IC_50_ value of greater than 50 *μ*g/mL, 15–50 *μ*g/mL, or less than 15 *μ*g/mL indicates inactive, weak, or good antiplasmodial activity, respectively. Thus, AML (IC_50_ = 5.0 ± 0.08 *μ*g/mL) produced good antiplasmodial activity. THBE and AMSBE (IC_50_ = 28.8 ± 0.16 and 24.5 ± 0.21 *μ*g/mL, respectively) (Table 3), produced weak in vitro antiplasmodial activity. However, APLE (IC_50_ = 110.1 ± 0.12 *μ*g/mL) was inactive. AMSBE and APLE were shown to demonstrate significant antimalaria activity in mice in vivo [20, 26]. However, these extracts were inactive against *P. falciparum* in vitro. This suggests that the extracts act as prodrugs in vivo where their metabolites might be responsible for their observed antimalaria action.

Furthermore, the extracts produced antitrypanosomal activity with IC_50_ values of 13.6 ± 0.21–53.0 ± 0.05 *μ*g/mL and that of the reference drug (DA) was 1.1 ± 1.13 *μ*g/mL (Table 3). The extracts can be arranged in increasing order of antitrypanosomal activity as follows: APLE < AMSBE < AML < THBE. Antitrypanosomal activity of the medicinal plant extract is classified as good, weak, or inactive when IC_50_ values of less than 8.0 *μ*g/mL ranging from 8.1–25.0 *μ*g/mL or greater than 25 *μ*g/mL are, respectively, obtained [27]. Therefore, three out of the four extracts in this study (AMSBE, AML, and THBE) produced weak antitrypanosomal activity (IC_50_ = 11.0 ± 0.09–19.9 ± 0.15 *μ*g/mL). However, APLE (IC_50_ = 53.0 ± 0.05 *μ*g/mL) was inactive.

The most important parameter in the cytotoxic test is the IC_50_ value that is defined as the concentration of a test substance, which can inhibit the viability of 50% of a cell under specific test conditions. IC_50_ values are used to measure the toxicity or safety of chemical agents in cytotoxic studies using cell lines. The smaller the IC_50_ value of a substance, the more toxic is the substance. Conversely, the higher the IC_50_ value of a test substance, the safer/less toxic is that substance [1]. In this study, AMSBE, AML, and THBE gave IC_50_ values of 98.5 ± 0.24–112.0 ± 0.32 *μ*g/mL which are relatively high. Hence, they are nontoxic (Table 3). APLE and the reference drug DA gave somewhat low IC_50_ values of 76.5 ± 0.22 and 74.1 ± 0.31 *μ*g/mL, respectively (Table 3). This makes them relatively toxic.

### 3.4. Selectivity Index (SI) of the Extracts

The activity and toxicity profiles in the parasites and macrophages resulted in the list of selectivity profiles are shown in Table 4.

The SI is the ratio of the IC_50_ value in mice macrophage RAW 264.7 to the IC_50_ value in the respective parasite (*P. falciparum* or *T. brucei*). The higher SI of a substance suggests the likelihood that the substance could produce innocuous treatment. And the smaller the SI of a substance, the more likely it is for that substance to produce ineffective and harmful treatment. The results obtained from this study, therefore, indicates that AML (antiplasmodial SI = 21.9) and THBE (antitrypanosomal SI = 21.9) recorded the highest SI against *P. falciparum* and *T. brucei,* respectively, among the extracts as the most promising in vitro antiplasmodial and antitrypanosomal agents, respectively. APLE emerged as the least promising antitrypanosomal and antiplasmodial agent since it has the least SI of 1.4 and 0.7, respectively, in both tests. It was suggested that a substance with an SI value <2 might produce wide-ranging toxicity [43]. Therefore, the extracts are safe in vitro except for APLE that could be classified as generally toxic in vitro.

The phytochemical analysis results showed that only AML and THBE have flavonoids present in them, whereas three to four other constituents were common to all the extracts. AML and THBE are the most active extracts in this study. This indicates that flavonoids may be responsible for the antiplasmodial and antitrypanosomal activity demonstrated by the two extracts in this study. Flavonoids such as apigenin, genkwanin, scutellarein, and diosmetin were reported to produce antitrypanosomal activity against *T. brucei* in vitro with IC_50_ of 5.1, 8.0, 4.6, and 6.1 *μ*g/mL, respectively [44].

## 4. Conclusion

The results from this study indicate that AML possessed good antiplasmodial activity, weak antitrypanosomal activity, andhighest SI among the extracts in the antiplasmodial test. THBE, also, produced good antitrypanosomal activity with the highest SI of 10.2 among the extracts. None of the extracts showedcytotoxic effect in the RAW 264.7 macrophage cell line except for APLE which was moderately cytotoxic and also produced the least SI in both antitrypanosomal and antiplasmodial tests. These results suggest that AML and THBE could offer safe and alternative therapy for malaria and trypanosomiasis. This is the first study to report the antitrypanosomal and in vitro antiplasmodial activities of these four plants. The activity of AML and THBE may be due to their chemical constituents such as flavonoids, which were absent in the other extracts. The AML and THBE could also be fractionated to isolate their constituents for preclinical, antiplasmodial, and antitrypanosomal studies.

## Figures and Tables

**Table 1:** The yield and nature of solid extracts produced.

Extract	Weight of the extract (g)	Weight of the extract (g)	Yield (%w/w)	Color of the extract
AMSBE	200	41.29	20.65	Dark brown
AML	200	9.43	4.72	Green
THBE	200	43.76	21.88	Reddish-brown
APLE	200	31.56	15.78	Green

**Table 2:** Phytochemical screening results of the extracts.

Phytochemical constituent	AMSBE	AML	THBE	APLE
Saponins	Present	Present	Present	Present
Phenolic compounds	Present	Present	Present	Present
Free reducing sugars	Present	Present	Present	Present
Flavonoids	Absent	Present	Present	Absent
Phytosterols	Absent	Present	Present	Present
Alkaloids	Absent	Absent	Absent	Present
Antracenosides	Absent	Absent	Absent	Absent
Polyuronoids	Absent	Absent	Absent	Absent
Triterpenes	Absent	Absent	Absent	Absent
Cyanogenic glycosides	Absent	Absent	Absent	Absent

**Table 3:** IC_50_ of the extracts against *P. falciparum, T. brucei,* and RAW 264.7 cell line inhibitions.

Extracts	*P. falciparum*	*T. brucei*	Cytotoxicity
	IC_50_ (*µ*g/mL)		
AML	5.0 ± 0.08	13.6 ± 0.21	109.4 ± 0.12
APLE	110.1 ± 0.12	53.0 ± 0.05	76.5 ± 0.22
THBE	28.8 ± 0.16	11.0 ± 0.09	112.0 ± 0.32
AMSBE	24.5 ± 0.21	19.9 ± 0.15	98.5 ± 0.24
DA	—	1.1 ± 1.13	74.1 ± 0.31
CHL	0.146 ± 0.05	—	6.88 ± 0.32

**Table 4:** The selectivity index (SI) of the extracts.

Extracts	SI	
	*P. falciparum*	*T. brucei*
AML	21.9	8.0
APLE	0.7	1.4
THBE	3.9	10.2
AMSBE	4.0	4.9
DA	N/A	67.4
CHL	47.12	N/A

NA = not applicable.

## Data Availability

The data can be obtained from the corresponding author upon reasonable request.
